# Combining graph and flux-based structures to decipher phenotypic essential metabolites within metabolic networks

**DOI:** 10.7717/peerj.3860

**Published:** 2017-10-12

**Authors:** Julie Laniau, Clémence Frioux, Jacques Nicolas, Caroline Baroukh, Maria-Paz Cortes, Jeanne Got, Camille Trottier, Damien Eveillard, Anne Siegel

**Affiliations:** 1 Institut de Recherche en Informatique et Systèmes Aléatoires, Centre National de la Recherche Scientifique, Rennes, France; 2 DYLISS, Institut National de Recherche en Informatique et Automatique, Rennes, France; 3 Laboratoire des Interactions Plantes Micro-organismes, Institut National de la Recherche en Agonomie, Castanet-Tolosan, France; 4 Center of Mathematical Modelling, Universidad de Chile, Santiago, Chile; 5 Laboratoire des Sciences du Numérique de Nantes, Université de Nantes, Nantes, France

**Keywords:** Graph-based analysis, Constraint-based analysis, Metabolic networks, Answer Set Programming, Essential metabolite

## Abstract

**Background:**

The emergence of functions in biological systems is a long-standing issue that can now be addressed at the cell level with the emergence of high throughput technologies for genome sequencing and phenotyping. The reconstruction of complete metabolic networks for various organisms is a key outcome of the analysis of these data, giving access to a global view of cell functioning. The analysis of metabolic networks may be carried out by simply considering the architecture of the reaction network or by taking into account the stoichiometry of reactions. In both approaches, this analysis is generally centered on the outcome of the network and considers all metabolic compounds to be equivalent in this respect. As in the case of genes and reactions, about which the concept of essentiality has been developed, it seems, however, that some metabolites play crucial roles in system responses, due to the cell structure or the internal wiring of the metabolic network.

**Results:**

We propose a classification of metabolic compounds according to their capacity to influence the activation of targeted functions (generally the growth phenotype) in a cell. We generalize the concept of essentiality to metabolites and introduce the concept of the *phenotypic essential metabolite* (PEM) which influences the growth phenotype according to sustainability, producibility or optimal-efficiency criteria. We have developed and made available a tool, *Conquests*, which implements a method combining graph-based and flux-based analysis, two approaches that are usually considered separately. The identification of PEMs is made effective by using a logical programming approach.

**Conclusion:**

The exhaustive study of phenotypic essential metabolites in six genome-scale metabolic models suggests that the combination and the comparison of graph, stoichiometry and optimal flux-based criteria allows some features of the metabolic network functionality to be deciphered by focusing on a small number of compounds. By considering the best combination of both graph-based and flux-based techniques, the *Conquests* python package advocates for a broader use of these compounds both to facilitate network curation and to promote a precise understanding of metabolic phenotype.

## Introduction

Deciphering phenotypic features of organisms is a fundamental pursuit in Biology (Kauffman, 1992). In particular, the self-organization of biological systems has been modeled to emphasize them (Karsenti, 2008). Following the rise of high-throughput sequencing technologies, this question is today more pertinent than ever: genome sequences and their annotations are available for numerous organisms or communities of organisms, and bioinformatics protocols have gained in maturity so they are now able to reconstruct their metabolic networks from these data (Henry et al., 2010; Magnúsdóttir et al., 2017). These metabolic networks are of particular interest because they represent the first extensive phenotype obtained from genome-scale knowledge. Metabolic networks differentiate sets of biochemical reactions that are specific from those that are more general and widely distributed over a large phylogeny. Because of this broader interest, many tools for reconstructing metabolic networks have been developed (Feist et al., 2009). An extensive analysis of these tools has shown that good genome annotation is the key to network quality (Liberal & Pinney, 2013). This sensitivity to genome annotation is particularly strong for recently sequenced organisms. It is usually compensated for by extensive post-analysis and metabolism curation stages that combine several approaches. First, a careful analysis of *metabolic network topology* potentially enables manual curation (Jeong et al., 2000; Romero & Karp, 2001; Handorf, Ebenhoh & Heinrich, 2005). Once the network structure is judged satisfactory (Ebenhoh & Heinrich, 2003; Liberal & Pinney, 2013), other techniques are applied by considering additional constraints such as *mass-balance equilibrium (or stoichiometry) of internal compounds* (i.e., Elementary Flux Modes (Schuster, Dandekar & Fell, 1999), Flux Coupling Analysis (Burgard et al., 2004) or Minimal Cut Sets (Klamt & Gilles, 2004; Beurton-Aimar, Nguyen & Colombie, 2014)). These techniques based on network stoichiometry allow the overall metabolic network consistency to be checked at quasi-steady state conditions (Stelling et al., 2002) but remain computationally challenging (Acuña et al., 2010). Complementary to topology and stoichiometry analysis of the network structure, other optimal flux-based techniques promote the use of environmental knowledge. For instance, by considering upper and lower bounds of exchange fluxes, optimization techniques try to maximize an objective function usually represented by the biomass, resulting in either one (i.e., Flux Balance Analysis Orth, Thiele & Palsson, 2010a) or several (i.e., Flux Variability Analysis (Gudmundsson & Thiele, 2010)) solutions for the flux distribution within the network. As illustrated in Goldford et al. (2017), interplays between the topological, stoichiometric and optimal-efficiency analyses are highly relevant to elucidate the functioning of a metabolic network and appropriately model the growth phenotype. Indeed, the stoichiometric framework provides information about the cell’s growth capability, in relation to cell lethality and biomass producibility. The constraint-based modeling framework provides information about the cell’s ability to optimize its biomass production (or production of any targeted compound) production, in relation to synthetic biology. However, both formalisms assume that the cell is in a steady-state, and implicitly allow the self-production of several internal compounds through balanced cycles to ensure biomass production from nutrient import. In a non-stationary growth phase, however, the dilution of nutrients may impact on the dependency of metabolites on their own production. In this case, cell sustainability is appropriately modeled in a graph-based framework by the so-called concept of network expansion (Kruse & Ebenhöh, 2008; Handorf et al., 2008).

Notably, deciphering key reactions and compounds is a major objective of metabolic network curation as well as of the analyses of growth phenotypes at the sustainability (graph-based framework), producibility (stoichiometry framework) and optimal-efficiency (constraint-based modeling framework) scales. Whereas some compounds are involved in linear pathways and play very little role in system functioning (Zhukova & Sherman, 2015), others are involved in transport between organelles and cytoplasm compartments, and are keystones for understanding phenotypic features (Peres, Felicori & Molina, 2013; Mintz-Oron et al., 2012; Borenstein & Feldman, 2009). Potentially, these compounds play a similar role to co-factors in understanding metabolic networks. More generally, this observation advocates for a modular decomposition of the metabolic network that puts a strong emphasis on internal compounds rather than focusing on exchanges only. However, despite their great success, the above methods consider *a priori* that all reactions and metabolic compounds are equivalent, an assumption that ignores the various roles they play in systems response because of the cellular structure (Klitgord & Segrè, 2010).

To address this issue, many authors have focused on the concept of *essentiality*, mainly in the stoichiometric and constraint-based modeling frameworks. In the mass-balanced formalism, an *essential reaction* is one where its removal (e.g., an *essential gene* deletion) is lethal, in the sense that it prevents the system from growing according to the Flux Balance formalism (Winzeler et al., 1999; Edwards & Palsson, 2000; Duarte, Herrgård & Palsson, 2004; Palumbo et al., 2007; Samal et al., 2006). Notice that essentiality can be studied either in any growth media or in (conditional) specified media (Patil & Nielsen, 2005; Timmermans & Van Melderen, 2009; Manimaran, Hegde & Mande, 2009). More generally, a Minimal Cut Set (MCS) depicts a set of reactions whose removal is lethal but none of its subset is lethal (Klamt & Gilles, 2004; Beurton-Aimar, Nguyen & Colombie, 2014). A specific case of MCS is when all the reactions from the MCS share a common substrate. In this case, the shared metabolite is called an *essential metabolite* (Kim et al., 2007; Kim, Kim & Lee, 2010). In other words, *essential metabolites* are such that the removal of all (multiple) reactions consuming the metabolite in question is lethal whereas removing these reactions one by one is never lethal. This sheds lights on the dependency between parallel pathways starting in the same metabolite.

In the optimal flux-based framework, an *essential reaction* is one where its removal from the system leverages the optimal capability of the system to produce biomass according to the Flux Variability Formalism (Gudmundsson & Thiele, 2010). In this setting, the impact of a gene deletion on an essential reaction is either lethality (as in the stoichiometry-based definition) or a decrease in optimal biomass production, which provides information about redundant although less efficient pathways for ensuring growth. Essential reactions are the main constituents, although not the only ones, of the so-called high-flux backbone, which consists of all the reactions with the highest consumption and production flux associated to each metabolite of the network (Almaas et al., 2004; Fischer & Sauer, 2005). More generally, the so-called *shadow price* approach allows the testing of growth sensitivity to the constraint associated either with a given reaction or with a nutrient (Ramakrishna et al., 2001). From a dual point of view, approaches based on *reduced costs* model the impact of a nutrient flux modification on the fluxes of the metabolic network. This sheds light on compounds of interest in the optimal flux-based framework, such as the demand for co-factors and the supply of nutrients (Savinell & Palsson, 1992).

Two limitations arise from this short review of the methods targeting essentiality within metabolic network. First, the concept of essentiality has be studied mainly by focusing on reactions, apart of the study of essential metabolites in Kim et al. (2007), and the supply or demand of nutrients and co-factors with shadow price analyses (Savinell & Palsson, 1992). Second, no study has considered the graph-based level for investigating essentiality. Graph-based studies shed light on highly-connected metabolites, according to several metrics, such as degree (Patil & Nielsen, 2005) or more general centrality measures (Liberal & Pinney, 2013). However, it should be noted that these metrics do not take into account the system response but only the graph structure. This is pointed out by Samal et al. (2006), who shows that essential reactions (according to a stoichiometric criteria) are related to low degree metabolites. As a first step towards taking system response into account, *reporter metabolites* (Patil & Nielsen, 2005) represent hotspot metabolites with respect to several transcriptomic responses of a biological system. They pinpoint regulations either allowing homeostasis to be maintained or metabolite concentration to be adjusted to a new functioning state of the system. At the metabolic level, however, no concept of graph-based essentiality with respect to growth exists.

This paper studies the role of internal metabolic compounds with respect to the production of a targeted metabolite. Metabolic compounds are hence classified and compared according to their ability to influence the growth phenotype at the sustainability, producibility and optimal-efficiency modeling scales. Following such a classification, we propose to define metabolic compounds called *phenotypic essential metabolites (PEM)*, or *crossroads*, that cannot be removed (e.g., they are always necessary to produce a given biomass component). Three categories of phenotypic essential metabolites will then be further defined based on their deliverability: (i) *sustainability essential metabolites* (sustainability-PEM); (ii) *producibility essential metabolites* (producibility-PEM); and (iii) *optimal-efficiency essential metabolites* (optimal-efficiency-PEM). Sustainability essential metabolites are compounds that promote biomass production according to the graph-based network-expansion criteria (Handorf, Ebenhoh & Heinrich, 2005; Kruse & Ebenhöh, 2008; Handorf et al., 2008). Therefore, perturbing all genes of reactions for which they are a substrate may impact on cell sustainability. Producibility essential metabolites are compounds that promote biomass flux, according to mass-balance criteria and steady-state assumptions. They are either substrates of essential reactions as introduced in Patil & Nielsen (2005) or essential metabolites introduced in Kim et al. (2007). Therefore, perturbing all genes of reactions for which they are a substrate may impact of biomass producibility and lead to lethality. Finally, optimal-efficiency essential metabolites are compounds that trigger the optimal production of biomass flux. In particular, we focused our analysis on substrates of reaction carrying optimal fluxes (effect of single gene deletion), in order to shed light on the differences between the stoichiometric and optimal-flux modeling scales. From a computational viewpoint, identifying these compounds relies on the combination of both Logical Programming (by extending the approach of Schaub & Thiele (2009)), and state-of-the-art Mixed Integer Linear Problem (MILP) optimization-based approaches implemented in Flux Balance and Variability Analyses (Ebrahim et al., 2013). In this paper, we have implemented and made available the *Conquests* tool (Crossroad in metabOlic Networks from Stoichiometric and Topologic Studies). This tool combines both topological and flux-based features analysis, which are usually assumed to be three distinct approaches, graph, stoichiometry and optimal-flux based respectively.

For the sake of application, we have studied PEMs in six genome-scale metabolic networks, ranging from the highest standard (*i.e.*, most recent *E. coli* metabolic network) to medium standard metabolic networks (*i.e.*, metabolic networks of non-model organisms). Interestingly, the three classes of phenotypic essential metabolites do not completely overlap. We provide a dynamic interpretation of metabolic network structures via differences between the sustainability, producibility and optimal-efficiency PEMs. By considering the best combination of both graph-based and flux-based techniques, *Conquest* advocates for a broader use of these compounds both to facilitate network curation and to promote a precise understanding of metabolic phenotypes.

## Material and Methods

### Definition and properties

#### Metabolic network

**Figure 1:**
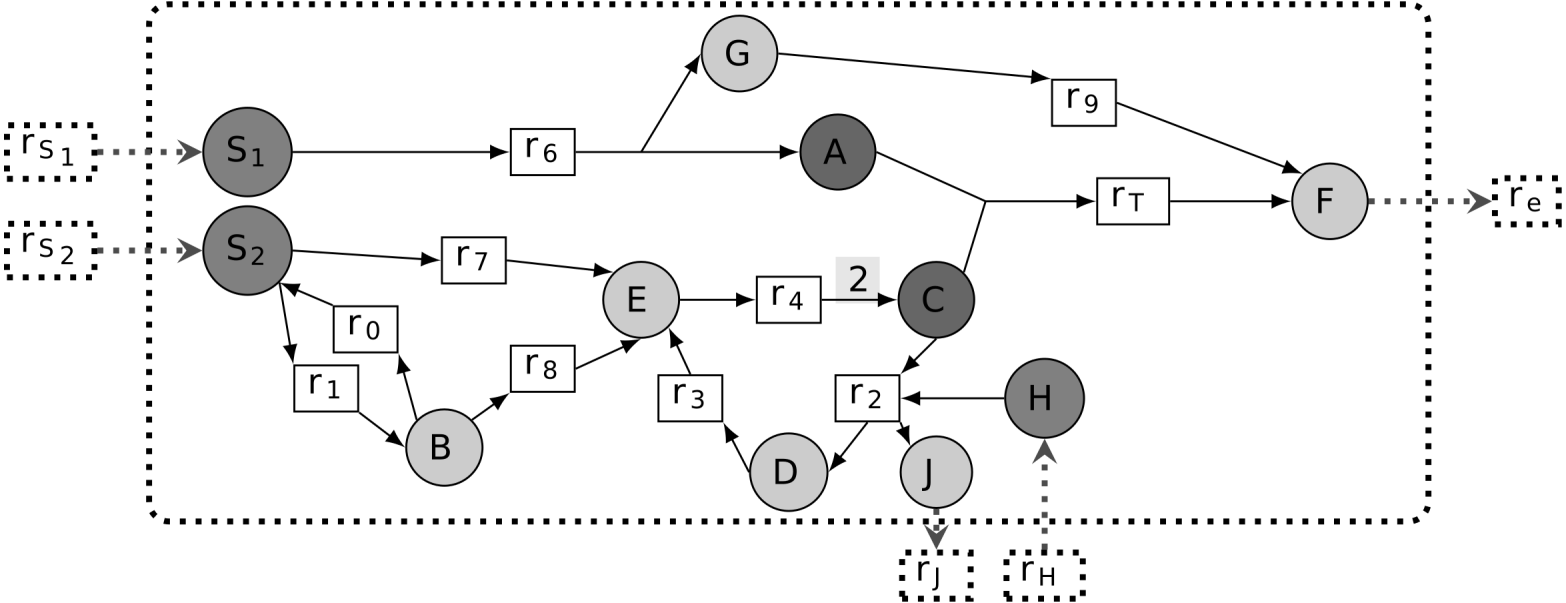
Representation of a metabolic network as a bipartite graph. All edge stoichiometry labels are equal to 1 on default, except for the edge from *r*_4_ to *C*: according to the label of *r*_4_, this reaction produces 2*C* from *E* and and therefore both feeds the *r*_2_, *r*_3_, *r*_4_ cycle and produces the extra *C* consumed by *r*_*T*_. In addition, the input and output of compounds *H* and *J* through *r*_2_ ensures mass conservation by the cycle. Reactions *r*_*S*_1__, *r*_*S*_2__ and *r*_*H*_ are boundary reactions because they do not show any incoming edges. The model seeds are {*S*_1_, *S*_2_, *H*} and *r*_*T*_ is the targeted reaction, because it allows biomass *F* production, by definition exported from the cellular compartment with *r*_*e*_ (*i.e.*, dashed line). The corresponding associated targeted metabolic compounds are then both *A* and *C*.

A *metabolic network* is commonly represented as a directed bipartite graph (*R*∪*M*, *E*), where *R* and *M* are sets of nodes standing for *reactions* and *metabolites*, respectively (see Fig. 1 for illustration). For any *r* ∈ *R*, we define *rcts*(*r*) = {*m* ∈ *M*∣(*m*, *r*) ∈ *E*} and *prds*(*r*) = {*m* ∈ *M*∣(*r*, *m*) ∈ *E*}. In other words, when (*m*, *r*) ∈ *E* or (*r*, *m*) ∈ *E* for *m* ∈ *M* and *r* ∈ *R*, the metabolite *m* is called a *reactant* or *product* of reaction *r*, respectively. Quantitatively, reactions are subject to *stoichiometry* for balancing the relative quantities of reactants and products. This can be captured by an *edge labeling* giving the stoichiometric coefficient of a reaction’s reactants and products, viz. *s*:*E* → ℚ, respectively. We designate (*R*∪*M*, *E*, *s*) a *stoichiometric metabolic network*.

We denote by $\mathcal{R}ev(M)$ the set of *reversible reactions* of the system. It corresponds to the set of reactions which can be associated with another reaction of the system with reverted reactants, products and stoichiometry. Formally, it is defined as follows: $\mathcal{R}ev(M)=\{r\in R\mid \exists {r}_{1}\in R,\forall m\in \mathit{rcts}(r),({r}_{1},m)\in E~\text{with}~s(m,r)=s({r}_{1},m)~\text{ and }~\forall m\in \mathit{prds}(r),(m,{r}_{1})\in E~\text{with}~s(r,m)=s(m,{r}_{1})\}$. See Fig. 1 for a reversible reaction illustration.

Each reaction *r* ∈ *R* is associated with a *metabolic flux value* (or activity rate) *v*_*r*_, a real variable confined by a upper bound *ub*_*r*_ ∈ ℝ^+^.

(1) \begin{eqnarray*}& 0\leq {v}_{r}\leq {\mathit{ub}}_{r} \forall r\in R.\end{eqnarray*}

#### Inputs and outputs of a metabolic network: set of seeds, boundary seeds and targeted reaction

We introduce the set *S*⊆*M* to model *initiation seeds or nutrients*, a set of metabolic compounds that are observed to be present in the system in its initial state. In addition, the graph structure includes a specific set of compounds whose production is intrinsically assumed to be activated by default. These *boundary compounds or boundary seeds* are defined as follows: *S*_*b*_(*G*) = {*m* ∈ *prds*(*r*)|∃*r* ∈ *R*, *rcts*(*r*) = ∅}⊆*M*. For the sake of clarity, we assume herein all boundary compounds to be seeds: *S*_*b*_(*G*) ⊂ *S*.

Its *targeted metabolic compounds* are hence assumed to be produced by the system. These are defined to be the reactants *rcts*(*r*_*T*_) of the target reaction (see Fig. 1).

#### Stoichiometry-based production of a targeted compound

The concept of activated products (or reactions) can be modeled on the basis of different paradigms. According to the state-of-the-art, the most widely used formalism for producibility is Flux Balance Analysis (FBA) (Orth, Thiele & Palsson, 2010).

In this paradigm, each reaction *r* is associated with a *metabolic flux value v*_*r*_, a real variable confined by Eq. (1). Flux distributions are formalized in terms of a system of equations relying on the stoichiometric coefficients of reactions. Reaction rates are governed by the *law of mass conservation* which assumes a steady state, *i.e.*, input and output rates of reactions consuming and producing a metabolite are balanced.

(2) \begin{eqnarray*}& \sum _{\begin{array}{@{}l@{}} \displaystyle (r,m)\in E \end{array}}s(r,m)\cdot {v}_{r}-\sum _{\begin{array}{@{}l@{}} \displaystyle (m,r)\in E \end{array}}s(m,r)\cdot {v}_{r}=0 \forall m\in M.\end{eqnarray*}

Definition 1 Let (*R*∪*M*, *E*, *s*) be a stoichiometric metabolic network. A reaction *r*_0_ is *stoichiometrically activated* if and only if the following holds:

\begin{eqnarray*}\exists \{{v}_{r},\,r\in R\}\subset {\mathbb{R}}^{+}\text{s.t.}{v}_{{r}_{0}}\gt 0~\text{and Eqs. (1), (2) hold}. \end{eqnarray*}

Worth noticing that from this definition, stoichiometrically activated reactions depend heavily on boundary compounds for which (2) is always satisfied and allows the values of fluxes to be initiated. For illustration, in Fig. 1, assuming that *ub*(*r*_*S*_1__) > 0, *ub*(*r*_*S*_2__) > 0 and *ub*(*r*_*S*_*H*__) > 0 implies that all reactions, including *r*_*T*_, are stoichiometrically activated. On the contrary, in the absence of *S*_2_ (*i.e*, *ub*(*r*_*S*_2__) = 0), all reactions are stoichiometrically inactivated except *r*_7_ and *r*_8_. Indeed, applying the mass conservation law to *B* and *S*_2_, all flux distributions must satisfy *v*_0_ = *v*_1_ + *v*_7_, *v*_1_ = *v*_0_ + *v*_8_ with *v*_7_, *v*_8_ ≥ 0, which implies *v*_7_ = *v*_8_ = 0. In addition, the stoichiometry associated with the edge from *r*_4_ ensures that the cycle *r*_2_- *r*_3_- *r*_4_ is activated and that it produces extra quantities of *C* to feed the reaction *r*_*T*_. This stoichiometry over the reaction *r*_4_ is crucial to ensure that the cycle *r*_2_, *r*_3_, *r*_4_ is not thermodynamically infeasible (Maranas & Zomorrodi, 2016), that is, a set of reactions that do not loop wth other entering or leaving metabolites. In addition, the role of compounds *H* and *J* in the reaction *r*_2_ is to provide an external input of matter to the cycle and avoid a second type of thermodynamic infeasibility.

#### Graph-based (topological) production of a targeted reaction

An alternative to stoichiometry-based modeling for activated reactions is graph-based modeling, by figuring out how metabolites can be transformed by chains of reactions using the graph topology. In this area a fundamental issue is to elucidate how cycles are activated according to the graph topology (De Figueiredo et al., 2009; Schaub & Thiele, 2009; Acuna et al., 2012). In the following, we focus on the most stringent semantics for graph-based production, originally introduced in Handorf, Ebenhoh & Heinrich (2005). These semantics were first shown to be of interest for identifying important metabolites in the context of network evolution (Raymond & Segrè, 2006; Goldford et al., 2017), and for inferring minimal nutrient requirements (Handorf et al., 2008). They have also also been used in the reconstruction of metabolic networks for non-model organisms (Romero & Karp, 2001; Handorf, Ebenhoh & Heinrich, 2005; Prigent et al., 2017; Frioux et al., 2017).

Because the semantics associated with reaction activation are based on graph topology, their definition is recursive. Given a metabolic network *G*, a reaction *r* ∈ *R* is *topologically activated* from a set of seeds *S* if all reactants in *rcts*(*r*), that is, the predecessors of *r* in the graph, are reachable from *S*. To deploy such a recursive definition, we say that, a metabolite *m* ∈ *M* is *topologically activated* from *S* if *m* ∈ *S* or if *m* ∈ *prds*(*r*) for some reaction *r* ∈ *R*, where all *m*′ ∈ *rcts*(*r*) are topologically activated from *S*. In other words, at least one predecessor of *m* in the graph is itself activated from *S*. The *scope* of *S* corresponds to the set of metabolites activated from the initial seeds of the network. Therefore, it is nothing other than the closure of the set of seeds on the hypergraph, as shown in several theoretical studies (Dittrich & Di Fenizio, 2007; Cottret et al., 2008).

More formally, the concept of topological activation is defined as follows.

Definition 2 Let (*R*∪*M*, *E*, *s*) be a stoichiometric metabolic network and let *S* be a set of seeds of the network such that *S*_*b*_(*G*) ⊂ *S*.

The *scope* of *S*, written Σ(*G*, *S*), is the closure of metabolites activated from *S*. It is defined as Σ(*G*, *S*) = ∪_*i*_*M*_*i*_ where *M*_0_ = *S* and ${M}_{i+1}={M}_{i}\cup \mathit{prds}(\{r\in \mathcal{R}\mid \mathit{rcts}(r)\subseteq {M}_{i}\})$.

A reaction *r*_0_ is *topologically activated from S* if and only if its reactants belong to the scope of the graph, that is *rcts*(*r*_0_) ⊂ Σ(*G*, *S*).

According to the toy example in Fig. 1, all metabolites are topologically activated from the set of seeds {*S*_1_, *S*_2_, *H*}, implying that *r*_*T*_ is topologically activated. Notice, however, that this property is not valid any more if we consider the seeds *S*_1_ and *S*_2_ independently. The set of reactions that are topologically activated from *S*_1_ is {*r*_6_, *r*_9_, *r*_*e*_} and the set of metabolites that are topologically activated from *S*_1_ is {*S*_1_, *A*, *G*, *F*}. Indeed, the only reaction topologically activated from the seed *S*_1_ is *r*_6_. Therefore, *G* and *A* are topologically activated from *S*_1_. By recursivity, we get that *r*_9_, hence *F* and *r*_*e*_ are activated. On the contrary, *C* cannot be activated from *S*_1_ only, implying that *r*_*T*_ is not activated. Similarly, the sets of reactions and metabolites that are topologically activated from {*S*_2_, *H*} are {*r*_0_, *r*_1_, *r*_2_, *r*_3_, *r*_4_, *r*_7_, *r*_8_} and {*S*_2_, *B*, *C*, *D*, *E*, *H*, *J*}. Therefore, *r*_*T*_ is not topologically activated from {*S*_2_}. Assuming that all *S*_1_, *S*_2_ and *H* are seeds, all reactions and compounds are topologically activated so that *r*_*T*_ is topologically activated from {*S*_1_, *S*_2_, *H*}.

### Implementation: the *Conquests* package

The computation of graph, stoichiometry and optimal-efficiency PEMs was performed by the Python package *Conquests* available at https://github.com/jlaniau/conquests. For the sake of application, each metabolic network was represented using its SBML file and incorporated into *Conquests* as an input, along with the identifier of a targeted reaction *r*_*T*_. The SBML file was then parsed with lxml Python package functions in order to identify the boundary seed compounds *S* and the list of substrates *T* of the targeted reaction. Sustainability-PEMs computation was performed using Answer Set Programming (ASP) and the pyASP Python package that calls upon the clingo4 grounder and solver. Finally, the CobraPy Python package (Ebrahim et al., 2013) was used to check whether the network is producing a flux for the targeted reaction *r*_*T*_. It was also used to perform Flux Variability Analysis (FVA) and return an interval [*min*_*v*_, *max*_*v*_] of values allowed for the fluxes *v*_*r*_ when studying an optimal targeted flux *v*_*r*_*T*__.

### Metabolic networks data

For the sake of application, several genome-scale metabolic networks were considered. *Escherichia coli* str. K-12 substr. MG1655 was analyzed via three of its publicly available models: *iJR*904 (Reed et al., 2003), *iAF*1260 (Feist et al., 2007) and *iJO*1366 (Orth et al., 2011), to depict the evolution of the metabolic networks of a single organism and mimic curations as proposed by cohorts of models of a model organism. Along with *E. coli*, the cyanobacterium *Synechocystis* sp. PCC 6803 (Knoop et al., 2013) was chosen as a complementary photosynthetic model to target complementary oxydo-reduction features that heterotrophic strains. Finally, other exotic organisms were selected: a biomining gamma-proteobacteria *Acidithiobacillus ferrooxidans*, str. Wenelen (Campodonico et al., 2016), and the eukaryota micro-alga *Tisochrysis lutea* (Baroukh et al., 2014). These last organisms were chosen for the sake of application of *Conquests* on less investigated networks. All the chosen organisms offer dedicated metabolic models that are functionally validated by means of both flux-based (positive rate for biomass production in FBA) and graph-based (reachability of biomass reactants) producibility assessments.

## Results

### A formal definition for phenotypic essential metabolites (PEM)

The goal of this section is to introduce several notions for metabolites of interest with respect to the metabolic network structure. We introduce three classes of phenotypic essential metabolites (PEM), corresponding to three different semantics for a metabolic network functionality.

We define the *pruning* of a metabolic network *G* = (*R*∪*M*, *E*, *s*) with respect to a metabolic compound *m* ∈ *M* as the subgraph of *G* in which all reactions having *m* as a substrate have been removed. More precisely, we introduce *prune*(*G*, *m*) = (*R*′∪*M*′, *E*′, *s*) such that *R*′ = *R*∖({*r* ∈ *R*∣*m* ∈ *rcts*(*r*)}∪{*r* ∈ *Rev*(*G*)∣*m* ∈ *prds*(*r*)}) and *E*′ = (*M* × *R*′∪*R*′ × *M*)∩*E*. In more biological settings, the network *prune*(*G*, *m*) is a new metabolic network where there are no more reactions that consume *m*.

In order to introduce the definition of phenotypic essential metabolites, we shall point out metabolic compounds that play an important role with respect to the activation of a specific targeted reaction (the biomass function in general).

Definition 3 **Phenotypic essential metabolites (PEM)**

Let *G* = (*R*∪*M*, *E*, *s*) be a metabolic network. Let *S* be a set of seeds such that *S*_*b*_(*G*) ⊂ *S*. Let *r*_*T*_ ∈ *R* be a targeted reaction.

A metabolic compound *m* ∈ *M* is called a *sustainability-PEM* if *m*⁄ ∈ *S*∪*rcts*(*r*_*T*_), *r*_*T*_ is topologically activated from *S* with respect to the network *G*, and *r*_*T*_ is not topologically activated from *S* with respect to the network *prune*(*G*, *m*).

A metabolic compound *m* ∈ *M* is called a *producibility-PEM* if *m*⁄ ∈ *S*∪*rcts*(*r*_*T*_), *r*_*T*_ is stoichiometrically activated from *S* with respect to the network *G*, and *r*_*T*_ is not stoichiometrically activated from *S* with respect to the network *prune*(*G*, *m*).

A metabolic compound *m* ∈ *M* is called an *optimal-efficiency-PEM* if *m* ∈ *rcts*(*r*_0_) where *r*_0_ ∈ *R* is a reaction such that

\begin{eqnarray*}& & \max \{{v}_{{r}_{T}}\mid \exists \{{v}_{r},\,r\in R\}\subset {\mathbb{R}}^{+}\text{s.t. Eqs. (1), (2) hold}\}\nonumber\\\displaystyle & & \gt \max \{{v}_{{r}_{T}}\mid \exists \{{v}_{r},\,r\in R\}\subset {\mathbb{R}}^{+}\text{s.t.}{{v}_{r}}_{0}=0 \text{and Eqs. (1), (2) hold}\}. \end{eqnarray*}

**Figure 2:**
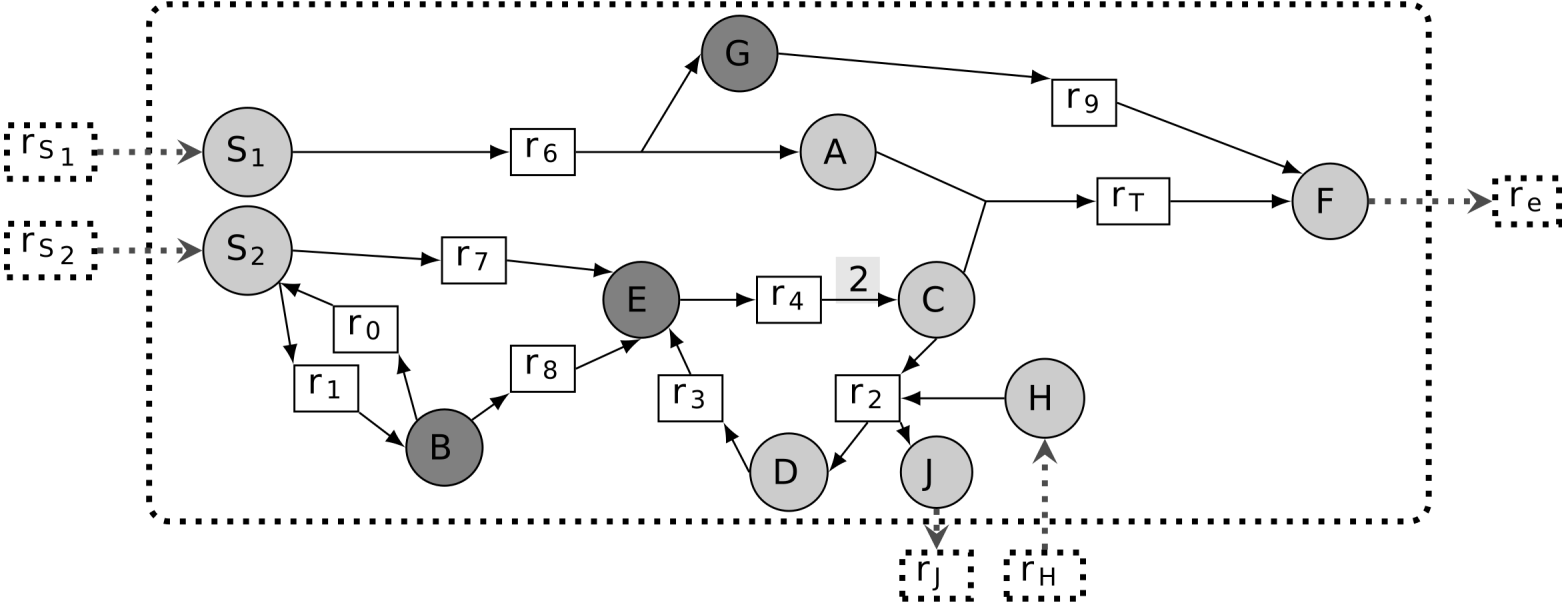
Different classes of phenotypic essential metabolites (PEM) in a metabolic network. The targeted reaction is *r*_*T*_. Node *E* has the three properties of being a sustainability-PEM, a producibility-PEM and an optimal-efficiency-PEM. Node *G* is both a producibility-PEM and an optimal-efficiency-PEM. Node *B* is an optimal-efficiency-PEM for certain values of flux upper-bounds.

According to Handorf, Ebenhoh & Heinrich (2005); Kruse & Ebenhöh (2008), a graph-based study of metabolic networks guided by the concept of scope is fundamental to modeling metabolic network sustainability, that is, the ability of a metabolic network to grow in a non-steady-state context. Based on this setting, a *sustainable essential metabolite m* is defined as being able to remove the system’s ability to activate a targeted reaction from the graph-based study of the metabolic network. In biological settings, as soon as all reactions consuming the compound *m* are removed from the network, there is no further possibility of joining the targeted reaction by recursively expanding the topology of the metabolic network. In other words, when *m* is a sustainability-PEM, the pruned network *prune*(*G*, *m*) contains a component of the targeted reaction, *m*_*T*_ ∈ *rcts*(*r*_*T*_), which no longer belongs to the scope of the seeds. Therefore, *m* is necessary to the production of *m*_*T*_ and to the activation of the targeted reaction. In the example shown in Fig. 2, *E* is the only sustainability-PEM, because its removal blocks activation of *C*, a reactant of the biomass reaction *r*_*T*_.

When the stoichiometry of a network is available, it becomes possible to modify the previous definition of sustainability-PEM by introducing flux constraints. The thinking behind this concept is that removing a metabolic compound may dramatically revert the stoichiometric balance of reactions and therefore prevent the target reaction flux from being activated. According to our Definition 3, a first case of producibility-PEM occurs for substrates of lethal reactions as introduced in Winzeler et al. (1999), also called essential reactions in Patil & Nielsen (2005). In this case, the considered compound is the substrate of a reaction in the network whose removal leads to lethality. A second case of producibility-PEM corresponds to the case when removing all reactions which consume the compound leads to lethality whereas the removal of any single reaction is not lethal. This correspond to the concept of *essential metabolites* introduced in Kim et al. (2007). In Fig. 2, *E* is a sustainability-PEM. In addition *G* is a producibility-PEM because removing the reaction *r*_9_ from the network means that any nonzero flux in *r*_6_ yields an accumulation of the compounds *G*.

As a final phenotypic essential metabolite concept, we focus on compounds impacting the optimal flux values associated with a metabolic network. From this point of view, the reactions of interest are the ones that ensure the maximum rate of the targeted reaction flux. They are called *essential reactions* in the Flux Variability Analysis (FVA) framework (Gudmundsson & Thiele, 2010). As a consequence, when essential reactions are removed from the network, the targeted reaction flux value decreases. Therefore, the system is sensitive to the flux through reactions having an optimal-efficient PEM as a substrate. Indeed, these reactions may have a negative shadow price on the targeted reaction flux (Ramakrishna et al., 2001; Savinell & Palsson, 1992).

Let us point out that essential reactions according to the optimal-efficiency criteria (FVA formalism) may not be essential (i.e., lethal) according to a producibility criteria. Indeed, in the FVA formalism, essential reactions are defined to be obligatory for the optimal production of biomass: they have a strictly positive flux in any flux distribution which optimizes the biomass production. Therefore, removing such a reaction from the network does not imply that the biomass cannot be produced. On the contrary, there may exist a non-optimal flux distribution which does not require the considered reaction and still allows the biomass to be produced. In Fig. 2, *E* is an optimal-efficiency-PEM. Assuming that the upper bounds of *v*_1_ and *v*_8_ are higher than the upper bound of *v*_7_, *B* also becomes an optimal-efficiency PEM since the optimal flux distribution to produce the targeted reaction *r*_*T*_ preferentially enables reactions *r*_1_ and *r*_8_.

One should notice that the definition of optimal-efficiency PEMs is slightly different from the definition of sustainability and producibility PEMs. The latter considers the case when all the reactions which consume a PEM lead to a change of phenotype (either sustainability or biomass production) whereas optimal-efficiency PEMs are restricted to the case when the considered compounds are the substrates of a single reaction whose removal leads to a change of phenotype (optimal biomass production). The motivation for such a difference is that using a similar definition for the three types of phenotypes would have introduced causalities between the PEMs: a producibility-PEM would have always been an optimal-efficiency PEM. On the contrary, as we will detail it in several case-studies, our definition of PEMs implies that there is no causality between the different concepts, increasing the relevance of comparing them.

### Efficient computation of sustainability-PEMs with a logic programming approach

The sustainability-PEM property is purely combinatorial. The main difficulty in identifying compounds that have this property is that the set of metabolites and reactions that are activated from a set of seeds within a network has to be computed recursively. In order to improve efficiency and avoid a separate computation on each pruned network, we introduced a logic programming approach to model the full set of constraints satisfied by the set of sustainability-PEMs and allow their identification in a single run of the program, as a refinement of the network expansion and scope logical modeling introduced in Schaub & Thiele (2009) and Collet et al. (2013).

In practice, the sustainability-PEM computation was performed using a logic programming approach known as Answer Set Programming (ASP) (Gebser et al., 2012). It is a declarative approach oriented toward combinatorial (optimization) problem-solving and knowledge processing. ASP combines both a high level modeling language with high performance solving engines with the result that the focus is on the specification of a problem rather than on the algorithmic part. The basic idea of ASP is to express a problem as a set of logical rules (clauses). Problem solutions appear as particular logical models (so-called stable models or answer sets) of this set. Modern ASP solvers like clasp (Gebser et al., 2014) support various combinations of reasoning modes, among them, regular and projective enumeration, intersection and union.

An ASP program consists of Prolog-like rules *h* :- *b*_1_, …, *b*_*m*_, *notb*_*m*+1_, …, *notb*_*n*_, where each *b*_*i*_ and *h* are literals and *not* stands for *default negation*. Mainly, each literal is a predicate whose arguments can be constant atoms or variables over a finite domain. Constants start with a lowercase letter, variables start with an uppercase letter or an underscore (don’t-care variables). The rule states that the head *h* is proved to be true (*h* is in an answer set) if the body of the rule is satisfied, i.e., *b*_1_, …, *b*_*m*_ are true and it cannot be proved that *b*_*m*+1_, …, *b*_*n*_ are true.

If the body is empty, *h* is a fact. They are used here to represent the input network. Substrates and products of a reaction are represented by facts reactant(M,R). and product(M,R)., where M and R denote metabolite and rule names. For reversible reactions, two rules are generated. We use facts seed(M). to represent seed metabolites and target(R). to represent the target reaction.

The recursive definition of metabolites in the *scope* of the network is provided in listing 1. It is either a seed metabolite (line 1) or a product of a rule such that all its reactants are in the scope of the network (line 2). This second line uses a notation *p*:*q*, which is satisfied if *p* is true for all possible *q*.

 
 
   Listing 1: Computing metabolites in the scope of a network 
  _______________________________________________________________________________________ 
1 scope (M ):− seed (M). 
2 scope (M2):− product (M2,R), scope (M1) : reactant (M1,R). 
  _______________________________________________________________________________________    

The definition of the pruned graph with respect to a metabolite is provided in listing 2. First, metabolites used for pruning are defined as reactants of the network scope. They are neither seeds nor reactants of a target reaction (line 1). Then reactions R that belong to the pruned network with respect to one of these metabolites M are represented by predicate pruned(R,M). They are all reactions of the network that are not using M as substrate.

 
 
                        Listing 2: Computing pruned graphs 
  _______________________________________________________________________________________ 
1 prune (M):− scope (M) ,   reactant (M,R) ,  not  seed (M) ,  not  target (R) . 
2 
3 pruned (R,M) :− prune (M) , reactant (_,R) , not reactant (M,R) . 
  ________________________________________________________________________________________    

Finally, according to its definition, a sustainability-PEM must be necessary to produce at least one substrate of the targeted reaction. In other words, there exists a reactant of the targeted reaction which does not belong to the scope of the set of seeds in the network pruned with respect to the sustainability-PEM. To model this property in the ASP program (listing 3), the scope of the set of seeds in a network pruned with respect to metabolite PM is computed (lines 1–3). A sustainability-PEM C is a metabolite that is necessary to produce a substrate TM of a target reaction, the latter being a metabolite that is no longer in the scope of the network when pruned with respect to C (lines 5–6). The set of sustainability-PEMs is produced with respect to any such possible substrate (line 7).

 
 
                         Listing 3: Computing the sustainability-PEMs 
  _______________________________________________________________________________________ 
1 scope (M  ,PM):− seed (M) . 
2 scope (M2,PM):− product (M2,R) , pruned (R,PM) , 
3                 scope (M1,PM) : reactant (M1,R) . 
4 
5 sustainabilityPEM (C,TM):− target (R) , reactant (TM,R) , scope (TM) , 
6                    prune (C) , not scope (TM,C) 
7 sustainabilityPEM (C):− sustainabilityPEM (C,_) . 
  _________________________________________________________________________________________    

### Application to biological models

#### Structural properties of the set of phenotypic essential metabolites

##### The study of six genome-scale metabolic networks suggests that they contain in average a few hundred PEMs.

As depicted in Table 1, our analysis evidences that the number of PEMs ranges from 113 to 423 in the studied examples, that is, from a small proportion (*E. Coli* metabolic networks, less that 13%) to a large proportion (most recent networks, more that 64%) of the network metabolites. Importantly, the number of PEMs does not depend linearly on the number of network metabolites. A possible interpretation is that networks with a small percentage of PEMs depict a relatively large number of pathways besides the primary metabolism of the organism that is related to the biomass modeling. However, it is expected that PEMs are mainly related to the growth phenotype and therefore to primary metabolism. On the contrary, most recent networks for less studied organisms focus on describing the primary metabolism, because fewer have been performed on these organisms than on model ones. This could suggest that the percentage of PEMs is an indicator of both the broad range of phenotypes encountered in the organism and the level of curation in its metabolic network.

**Table 1:** Number of phenotypic essential metabolites (PEMs) for six metabolic networks of Prokaryota and unicellular Eukaryota. The percentage in each cell depicts the ratio of metabolites with the property in question (column identifier) in the network in question (line identifier) out of the total number of metabolites in the network in question.

	Reactions	Metabolites	PEMs	Sustainability- PEMs	Producibility- PEMs	Optimal-efficiency- PEMs
*iJR*904	1075	904	113 (12.5%)	70 (7.7%)	65 (7.2%)	87 (9.6%)
*iAF*1260	2382	1967	177 (9.0%)	98 (5%)	105 (5.3%)	166 (8.4%)
*iJO*1366	2582	2129	150 (7.1%)	66 (3.1%)	67 (3.1%)	143 (6.7%)
*Synechocystis*	759	600	423 (70.5%)	325 (54.2%)	380 (63.3%)	375 (62.5%)
*A. ferrooxidans* st. Wenelen	620	579	376 (64.9%)	316 (54.6%)	340 (58.7%)	372 (64.2%)
*T. lutea*	316	324	210 (64.8%)	198 (61.1%)	208 (64.2%)	208 (64.2%)

#### All highly connected compounds are either PEMs, biomass components or seeds

Generic graph-based approaches usually point out the importance of *hubs*, that is, nodes with a high level of connectivity (Liberal & Pinney, 2013). In our context, the *degree of connectivity* of a metabolic compound *m* in a metabolic network (*R*∪*M*, *E*) was defined as *connectivity*(*m*) = card(*r* ∈ *R*∣*m* ∈ *rcts*(*r*)∪*prds*(*r*)). It depicts the number of reactions which either consume or produce the metabolite *m*. However, we notice that this definition does not take into account the *functioning role* of the considered compound with respect to the metabolic network, that is, its impact on the production of targeted metabolites. As a first analysis, we ordered the compounds of each metabolic network according to both their degree of connectivity and their role in the functioning of the network classified as PEM, biomass component, seed (that is, roughly, general system inputs from the extracellular compartment) or generic compounds. Our analysis confirmed that, as expected, regardless the considered network, all highly connected compounds are either a PEM, a biomass component or a seed (see Supplemental Information 1). Indeed, in the *iJR*904, *iJO*1366 and *iAF*1260 networks, the 14 compounds with the highest degrees of connectivity (from 70 to 1040) have such a role. The only exception is the periplasmic *H2O* of the *iJO*1366 network, a component which appears to play a redundant role in the network with cytoplasmic * H2O*. In the *A. ferrooxidans* and *T. lutea* networks, the 31 compounds with the highest degrees of connectivity (from 10 to 310) have such a role. We noticed however that the PEM concept encompasses a much larger set of compounds than the single family of highly connected compounds. This emphasizes that, in addition to the expected highly connected nodes, the PEM concept sheds light on metabolic compounds with a possibly impactful role in the production pathways of target compounds regardless their connectivity.

#### Putative role of sustainability, producibility and optimal-efficiency-PEMs

The distribution of sustainability, producibility and optimal-efficiency-PEMs is described in Table 1. According to this study, the most frequent PEM is the optimal-efficiency-PEM: depending on the metabolic network studied, from 77% to 99% of PEMs are optimal-efficiency-PEMs. Accordingly, for all networks, a small proportion of PEMs are not optimal-efficiency-PEMs. The number of sustainability and producibility-PEMs is fairly comparable: the ratio of producibility-PEMs ranges from 44.7% (*iJO*1366 network) to 99% (*A. ferrooxidans* network), whereas the ratio of sustainability-PEMs ranges from 46% (*iJO*1366 network) to 86% (*T. lutea* network). Similarly as before, for all networks, some PEMs are neither sustainability nor producibility-PEMs. This analysis confirms that the three classes of PEMs are complementary and must be considered together to capture the full complexity of the functioning of the network.

**Figure 3:**
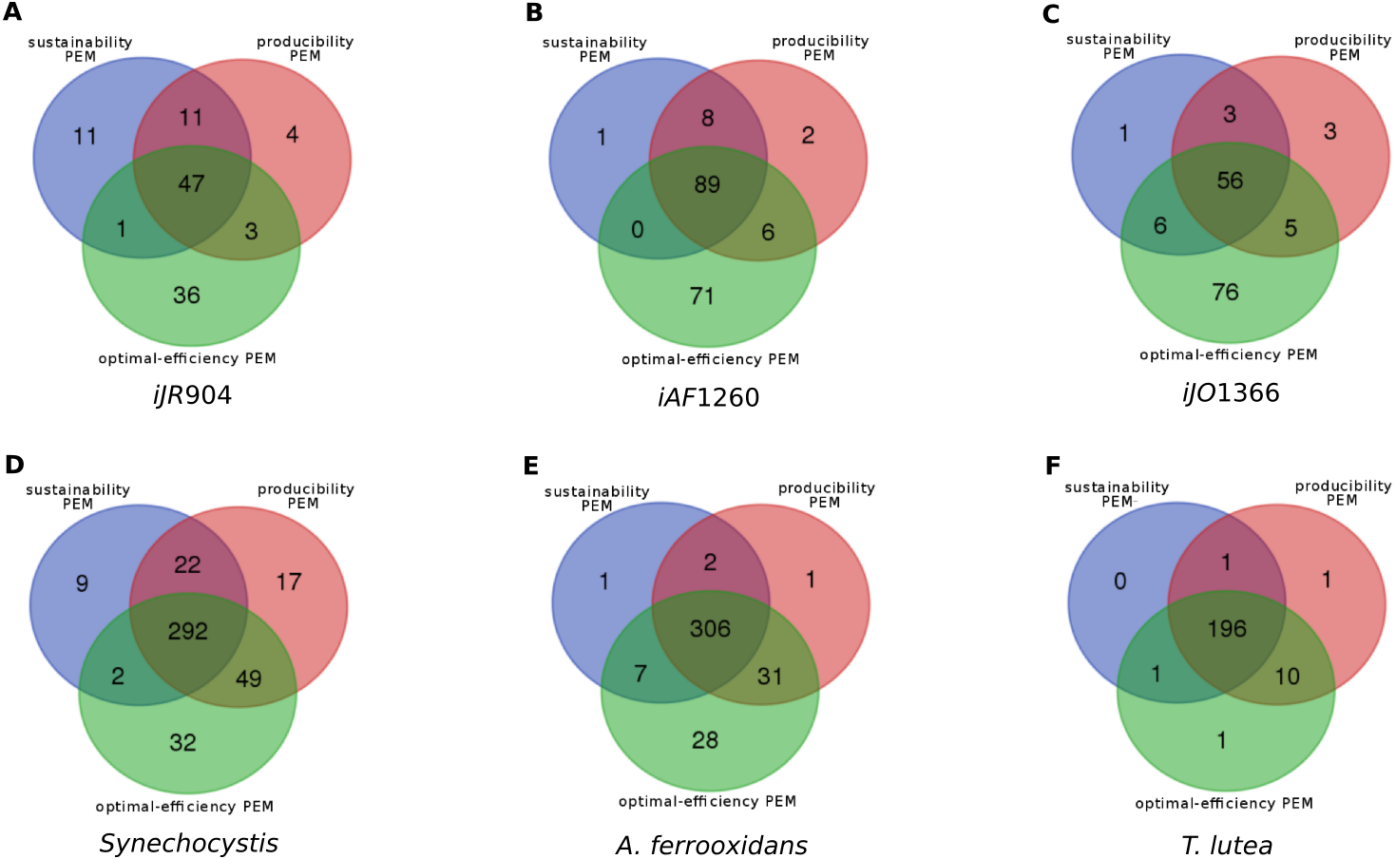
Overlaps between the sets of sustainability-PEMs (blue), producibility-PEMs (red) and optimal-efficiency-PEMs (green) for six metabolic networks shown in Venn diagrams. (A) E. Coli *iJR*904 (B) E. Coli *iAF*1260 (C) E. Coli *iJO*1366 (D) *Synechocystis* (E) *Acidithiobacilius ferrooxidans* str. Wenelen (F) *Tisochrysis lutea*.

Overlaps between the different classes of compounds for the six metabolic networks are shown in Fig. 3. At least 26% (and up to 93%) of the network PEMs are sustainability, producibility and optimal-efficiency-PEMs simultaneously. Comparing compounds which are sustainability, producibility and optimal-efficiency-PEMs in the *iJR*904, *iJO*1366 and *iAF*1260 networks highlights the fact that 20 metabolic compounds satisfy these three properties in all the three networks, including the highly connected nodes *udp*, *h*, *phosphate* and *phosphoenolpyruvate*. These four compounds can therefore be viewed as the major biomass production regulators of E. coli networks in the graph, stoichiometry and optimal flux-based formalisms. Surprisingly, the other 16 PEMs in the common skeleton of the *iJR*904, *iJO*1366 and *iAF*1260 networks have a low degree of connectivity in the network (from 2 to 7). These compounds are *Chorismate, DTDP, ahdt, Dihydroneopterin, Dephospho-CoA, 1,2-Dihydronaphthalene-1,2-diol, 6-hydroxymethyl-dihydropterin pyrophosphate, 4-amino-4-deoxychorismate, Dihydroneopterin monophosphate, 6-hydroxymethyl dihydropterin, N-((R)-4-Phosphopantothenoyl)-L-cysteine, 6-hydroxymethyl dihydropterin, 5-O-(1-Carboxyvinyl)-3-phosphoshikimate, D-4′-Phosphopantothenate, Pantetheine 4′-phosphate*. One interpretation is that these metabolites may refer to structural compounds of the network necessary to produce the biomass and may constitute a *skeleton* of the network structure regarding the biomass production.

#### Network redundancies

#### Sustainability and producibility-PEMs that are not optimal-efficiency-PEMs depict network redundancies.

**Figure 4:**
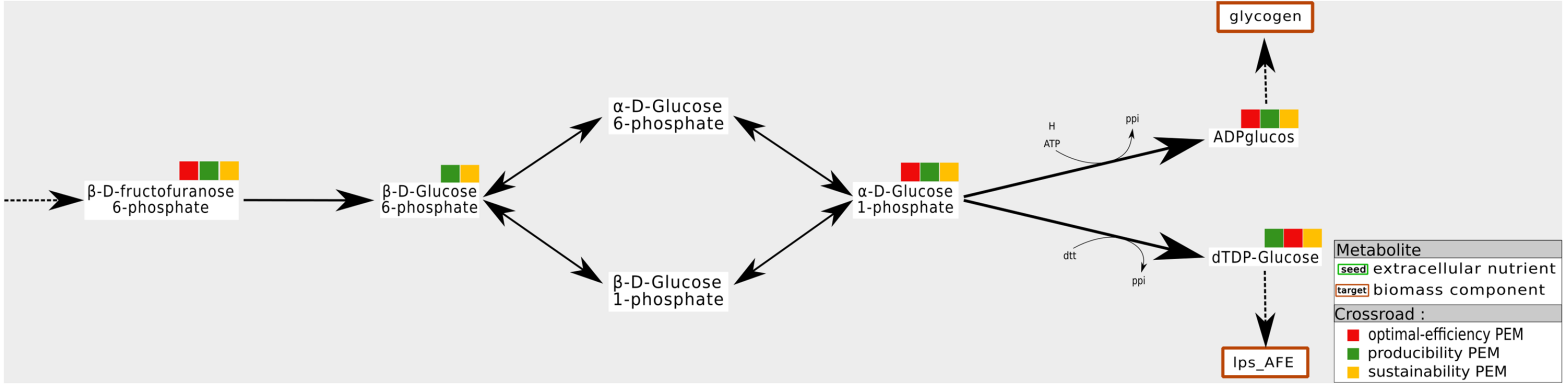
Network redundancies: example of sustainability and producibility-PEMs that are not optimal-efficiency PEMs. In the *A. ferrooxidans* network, the production of *glycogen* and *lps_AFE* can be handled by two alternative pairs of reactions, either through *α-g6p* (*α*-D-Glucose-6-phosphate) or through *β-g1p* (*β*-D-1-phosphate). *β-g6p* (*β*-D-Glucose-6-phosphate) is not an optimal-efficient PEM because removing any of the two reactions which consuming it has no impact on the optimal production of the biomass components. On the contrary, removing both reactions, as allowed by Definition 3 for sustainability and producibility PEMs, implies that the biomass components cannot be produced. Therefore, *β-g6p* is a sustainability and a producibility PEM.

This situation occurs when a compound is required to produce a biomass component from the stoichiometry and graph-based viewpoints. All the reactions consuming this component can be removed without impacting the optimal biomass growth rate. One interpretation of this is that this type of compounds is the substrate of two pathways, both equally capable of producing the targeted biomass component. An example is shown in Fig. 4. In the *A. ferrooxidans* network, the compound *g1p* is a precursor to *glycogen* and *lps_AFE*, two biomass components. Their production is ensured by a pathway starting from *β*-*f6p* and *β*-*g6p*. They can be transformed into either *α*-*g6p* or in *β*-*g1p*. No other pathway can produce these targets. Therefore, *β*-*g6p* is a both a sustainability and a producibility-PEM, but it is not an optimal-efficiency PEM.

Among the six metabolic networks studied, this situation is not frequently observed in the *iJO*1366, *A. ferrooxidans* and *T. lutea* networks. In the *Synecchocystis*, *iAF* 1260 and *iJR*904 networks this situation occurs from eight to 22 times. In particular, the relatively high number of such PEMs in the Synecchocystis network suggests that its optimal functioning is not highly constrained. This could be explained by the presence of a relatively high number of export fluxes (this has already been observed when studying PEMs that are producibility-PEMs only) which generate redundancies with intracellular pathways.

#### Optimal-efficiency-PEMs that are neither sustainability nor producibility-PEMs shed light on optimal flux-distributions among alternative pathways.

These PEMs are required to produce optimal biomass. Removing all reactions for which this PEM is a substrate does not prevent the biomass from being stoichiometrically and topologically activated. For instance, in the *Synecchocystis* network, the removal of the reactions for which *Cytosolic O-Phospho-L-serine* is a substrate causes the maximal biomass growth rate to decrease from 47.5 to 41.1 mmol/gDW/h.

**Figure 5:**
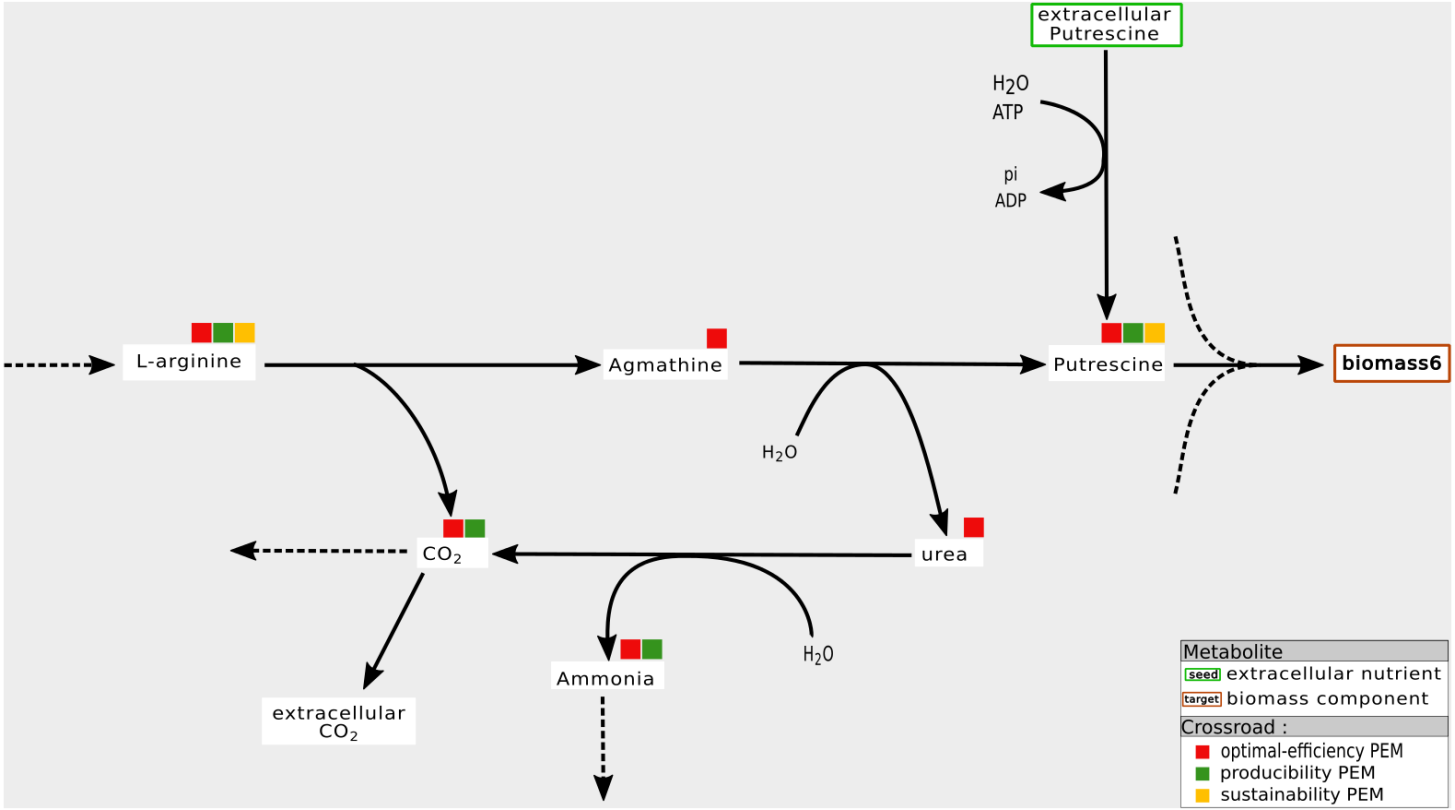
Optimal choice of pathways: example of an optimal-efficiency-PEM that is neither a sustainability nor a producibility-PEM. In the *Synecchocystis* network, the production of *Putrecine* is assured by a pathway initiated in *L-arginine* at the expense of a direct alternative pathway from extra-cellular *Putrecine*. One interpretation is that the *L-arginine* pathway also allows the production of *Ammonia*. In this context, *urea* and *agmathine* are optimal-efficiency-PEMs but neither sustainability-PEMs nor producibility-PEMs.

Our interpretation is that this mainly occurs when biomass components can be produced by at least two distinct pathways and when one of them is favored by the system on the basis of optimal flux-based analysis. An example is shown in Fig. 5. In the *Synecchocystis* network, the cytosolic *putrescine* is a precursor of the biomass components. In this network, two pathways produce this compound: one of them drives an import flux of *extracellular putrescine* and the other, an internal one, drives a pathway involving *L-arginine* and *agmathine*. *CO2* is a co-product of the latter pathway. Its relationship with the production of coproduct causes the network to favor the *L-arginine* pathway for producing *putrescine* in order to optimize the biomass growth rate. Therefore, *agmathine* and *putrescine* are optimal-efficiency-PEMs. However, they are neither sustainability and producibility-PEMs because they are not required for the graph-based and the stoichiometry-based productions of *putrescine*.

In the six networks studied in this paper, optimal-efficiency-PEMs that are neither sustainability nor producibility-PEMs are the most frequent PEMs after PEMs that are sustainability, producibility and optimal-efficiency-PEMs simultaneously. In the *iJR*904, *iAF*1260 and *iJO*1366 networks, between 32% and 50% PEMs have this property. The most recent *Synecchocystis* and *T. lutea* networks includes around 10% of such PEMs. The exception is the *A. ferrooxidans* network with very few differences between the graph, stoichiometry and optimal flux-based formalisms.

#### Dependency of pathway activation on the initial state of the cell

##### Sustainability-PEMs that are neither producibility nor optimal-efficiency-PEMs can provide information about the dependency of pathways on initial cell state.

A PEM which satisfies only the topology-based criteria is a necessary compound for the topological activation of a biomass component. However, this compound is unnecessary for producing the corresponding biomass component from a stoichiometric viewpoint. For instance, according to graph-based criteria, in the *iJO*1366 network, the cytosolic *thiamine* is necessary for producing the *thiamine diphosphate*, a biomass component. Indeed, if we prune the metabolic network by removing all the reactions whose substrate is cytosolic *thiamine*, the *thiamine diphosphate* is no longer topologically activated. On the contrary, in the pruned graph, the growth rate maximal value (the biomass reaction) is unchanged compared to the growth rate of the initial network (11.747 mmol/gDW/h). This apparent paradox is explained mainly by the dynamical assumptions underlying the stoichiometric and topological activation semantics. We assert that these different semantics impact heavily on the interpretation of a cycle functioning with respect to the dependency of metabolites on their own production (Kruse & Ebenhöh, 2008).

**Figure 6:**
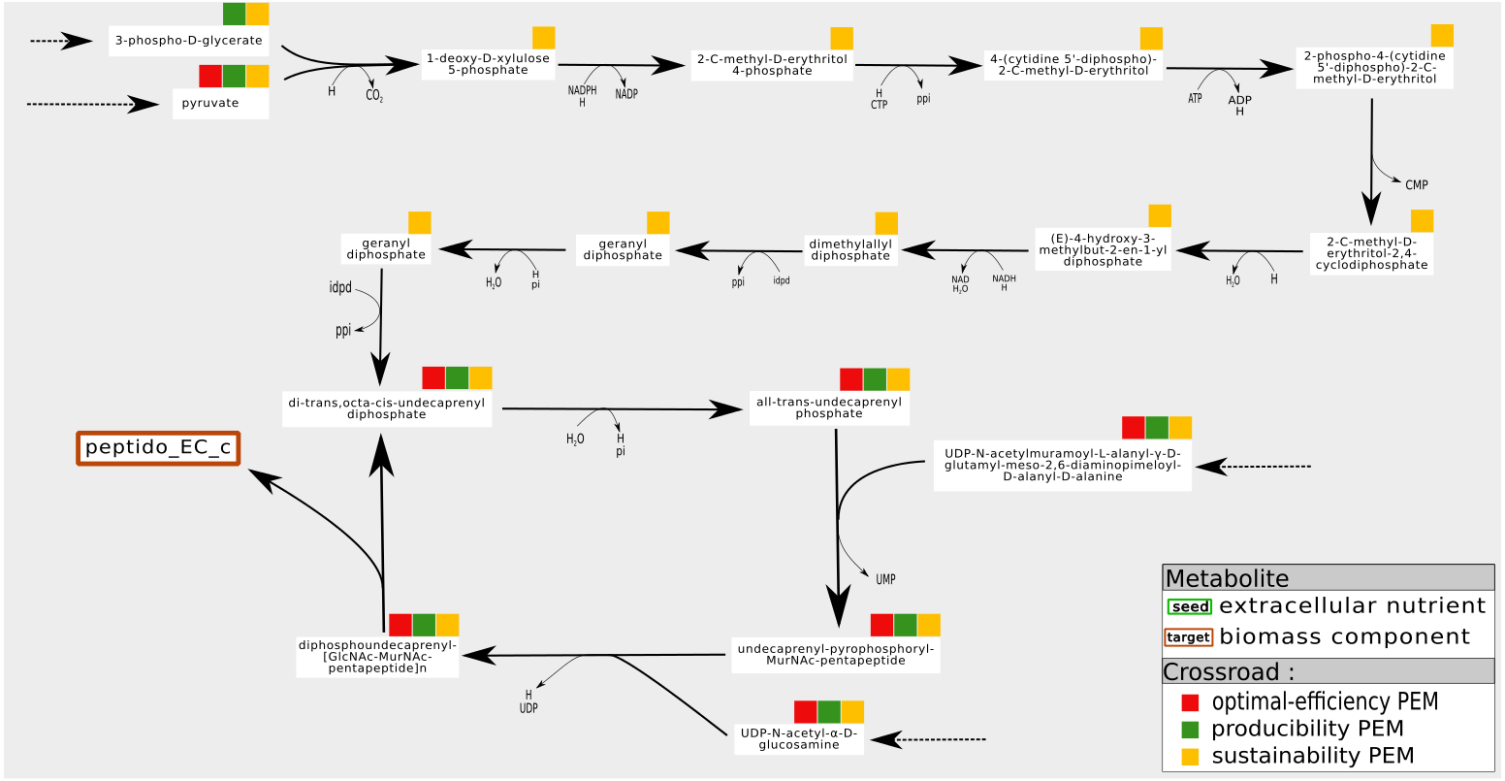
Pathways of which the activation is dependent on the initial state of the cell. Example of a sustainability-PEM that is neither a producibility-PEM nor an optimal-efficiency-PEM. In the *iJR*904 network, the production of the biomass component *peptido_EC* (Peptidoglycan subunit) is assured by a cycle from *di-trans,octa-cis-undecaprenyl diphosphate* to *diphospohdecaprenu-[GlcNAc-MurNAc-pentapeptide]n*. All the elements involved in the cycle are sustainability, producibility and optimal-efficiency-PEMs. The cycle is initiated by a pathway starting from *pyruvate* and composed of nine sustainability-PEMs that are neither producibility nor optimal-efficiency-PEMs. Indeed, at steady state, activation of the initiation pathway is not required for the production of an optimal biomass, although this pathway may be necessary to initiate the cycle when not in a steady state. The latter role in the functioning of the network is reflected by the sustainability property.

An example is shown in Fig. 6. In the *iJR*904 network, the compound *Peptidoglycan subunit*, a biomass component, is produced from the metabolite *uaagmda* which is involved in a cycle through the *Undecaprenyl diphosphate*. At steady state, stoichiometry-based analysis suggests that the production of * Peptidoglycan subunit* is assured by the self-activation of the cycle, since all the reactions in the cycle are essential to the optimal production of the biomass. However, this type of analysis implicitly assumes that at least one of the component of the cycle is present on the activation of the system, and that none of the components is degraded during system functioning, guaranteeing the system producibility at steady state. On the contrary, in the graph-based framework, the analysis requires cycles to be initiated from external pathways in order to be considered activated. This corresponds in particular to situations where the cells are growing and require external input to ensure their sustainability, as pointed out in Kruse & Ebenhöh (2008). In the example shown in Fig. 6, the cycle is initiated by a linear pathway of ten reactions starting from the * pyruvate* metabolite. Our analysis shows that nine compounds are required this linear pathway, from a graph-based point of view, to produce the targeted compound *Peptidoglycan subunit*. On the contrary, they are not needed to produce *Peptidoglycan subunit* at steady state (stoichiometric-based accessibility), because this compound is produced by a self-induced cycle, thus the reaction flux is equal to zero for optimal flux-based analysis. This analysis suggests that the production of *Peptidoglycan subunit* is dependent on *pyruvate*, in addition to its link with a self-activated loop.

To summarize, one interpretation of sustainability-PEMs that are neither producibility nor optimal-efficiency-PEMs is that it suggests the existence of a self-activated cycle in the flux-based analysis (producibility at steady state) and provides candidates for the initiation of cycles when the cell is not at steady state (sustainability during growth phases) (Kruse & Ebenhöh, 2008). However, with the six metabolic networks studied, this phenomenon is observed very infrequently: once in the *iAF*1260, *iJO*1366 and *T. lutea* networks, nine times in the *Synecchocystis* network and eleven times in the *iJR*904 network. This suggests a need for curation for networks with a medium number of sustainability-PEMs that are neither producibility nor optimal-efficiency-PEMs.

#### Sustainability and optimal-efficiency-PEMs that are not producibility-PEMs provide insights on internal cycles for production of non-optimal biomass production

This is a tricky situation, for when a compound has this property, it is the substrate of a reaction that is always activated when the cell produces an optimal biomass growth rate. The sustainability property means that, if this reaction is removed from the network, the targeted biomass component is no longer activated according to a graph-based criteria. Nevertheless, the biomass component still has the capability to be activated at the stoichiometric level, although with a lower growth rate. An alternative means of production of the targeted component exists, although this alternative pathway is not connected to the set of seeds from a graph-based viewpoint. For instance, in the *A. ferrooxidans* network, removing the reactions which consume *Alpha-D-Ribose_1-phosphate* causes the optimal biomass growth rate to decrease from 3.6 to 3.4 mmol/gDW/h. Therefore it is still producible although there is no longer a pathway from the set of seeds to the biomass reaction. One interpretation of this paradox is similar to the case shown in Fig. 5, concerning the self-activated loop.

**Figure 7:**
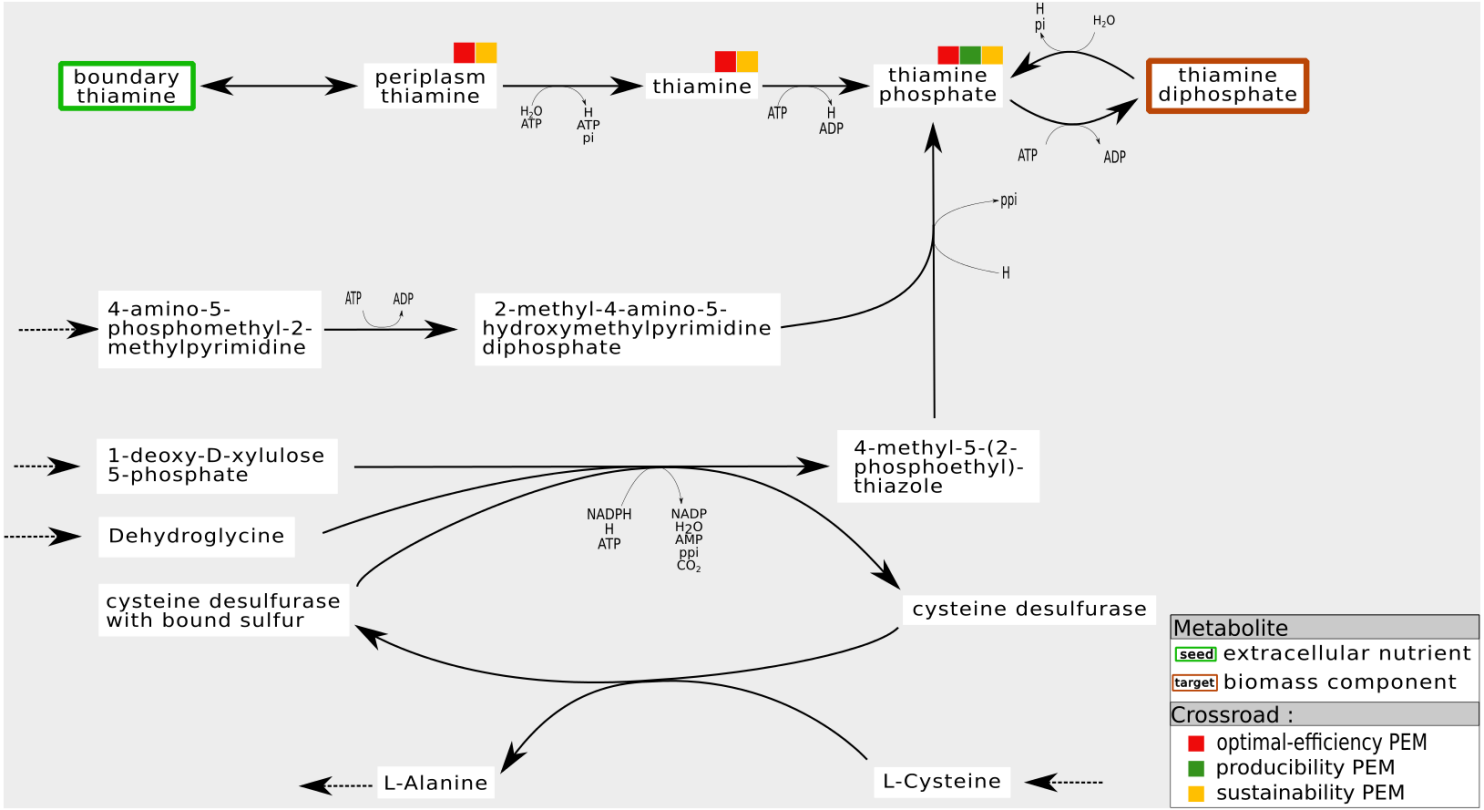
Internal cycles for non-optimal biomass production: example of sustainability and optimal-efficiency-PEMs that are not producibility-PEMs. In the *iJO*1366 network, removing the reaction consuming *Thiamin* to produce *Thiamin diphosphate* allows the system to activate an internal cycle. This internal cycle involves components that cannot be activated from the set of seeds according to graph-based criteria.

For instance, Fig. 7 shows a sub-network of the *iJO*1366 network. In the cytosol compartment, *Thiamin*, a PEM, is the only precursor of *Thiamin monophosphate*, a biomass component. Its production is assured by a pathway initiated by the set of seeds that imports *Thiamin* from periplasm to cytosol. Also, when the reaction from *Thiamin* to *Thiamin monophosphate* is removed from the network, the network has the ability to activate a cycle to produce *4-Methyl-5-(2-phosphoethyl)-thiazole* (*4mpetz*). Notice, however, that this cycle is not connected to the set of seeds since its components are not topologically activated. Therefore, its activation is heavily dependent on the presence of at least one of its components at the initiation of the cell dynamics.

Regarding the six genome-scale networks studied, this situation mainly occurs in the *iJO*1366 network (six times) and the *T. lutea* network (seven times). Therefore, both networks have the capacity to adapt themselves by activating internal mass-balanced cycles.

#### Control of system response through co-products equilibria

##### Producibility-PEMs that are neither sustainability nor optimal-efficiency PEMs may point out to the fine tuning of mass-balance equilibria.

Such PEMs have the ability to set the biomass flux rate to zero when all their associated substrate reactions are removed from the network even though none of the removed fluxes is required to produce the biomass in the initial network. In addition, the pruning operation does not remove the graph-based activation of the biomass component. An example in the *iAF*1260 network is the cytosolic *carbon dioxide*. Removing this metabolite from the network, with all the reactions that use it as a substrate, does not affect the graph-based activation of any of the target metabolites. However the growth rate is reduced from 9,021 to 0 mmol/gDW/h. The biomass component cannot be produced because of the non-balanced mass equation despite the fact that it is theoretically still able to be activated topologically.

**Figure 8:**
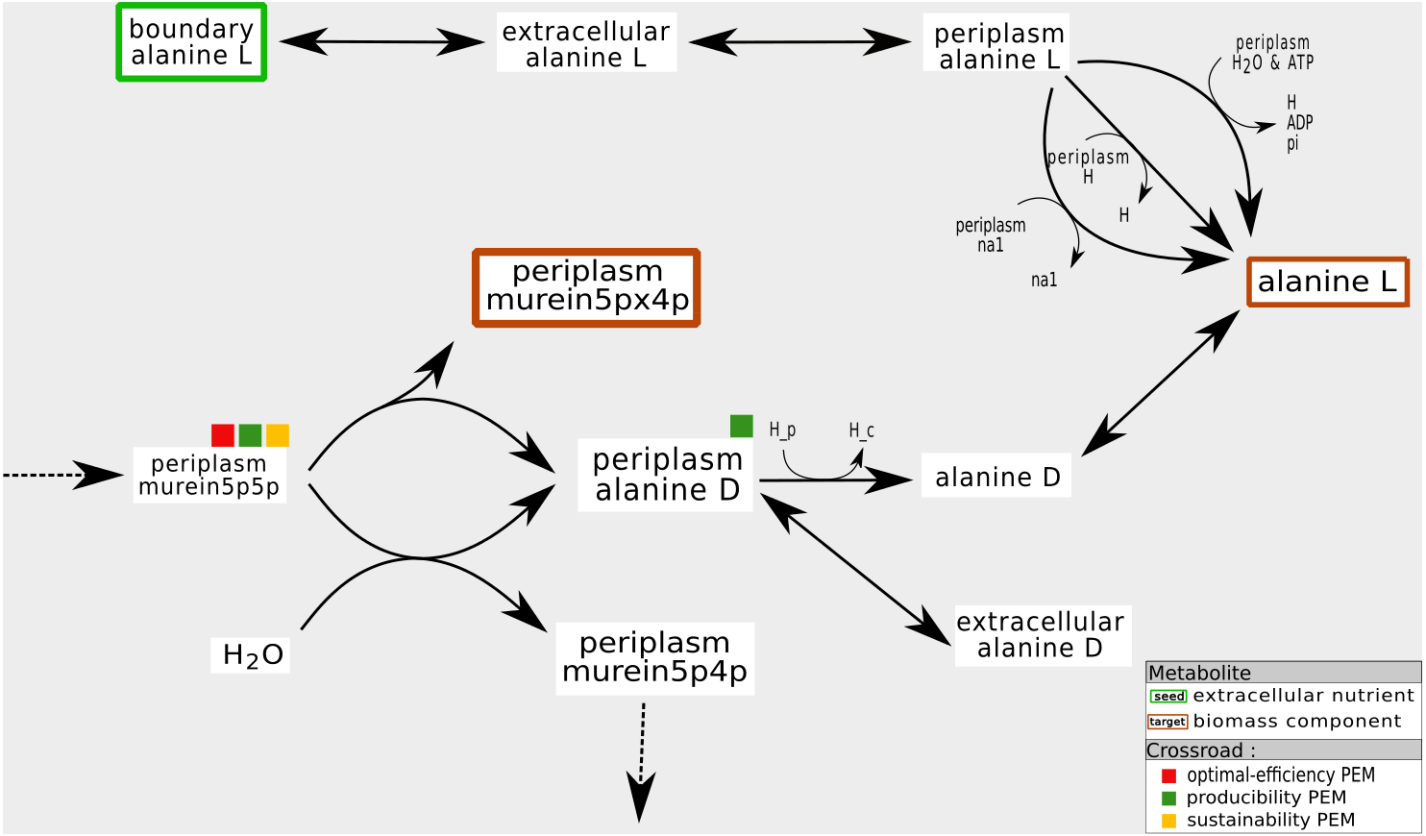
Fine tuning of mass-balance equilibria: example of a producibility-SEM that is neither a sustainability nor a optimal-efficiency-PEM. In the *iAF*1260 network, the production of the periplasmic *murein5px4p* (that is, the identifier of a biomass component in the BIGG database) is under the control of the export of its coproduct *D-Alanine* to the cytoplasm or to the external compartment. From the graph, stoichiometry and optimal-flux based viewpoints, the *murein5p5p* compound is required for the production of *murein5px4p*. On the other hand, the presence of *D-Alanine* ponly has an impact on the flux-based biomass production. If the *D-Alanine* export reaction is removed, it accumulates, which is a mechanism not allowed in a steady state.

As shown in Fig. 8, these compounds are mainly the co-products of essential reactions that need to be metabolized to ensure the biomass production. In the periplasmic compartment of the *iAF*1260 network (Fig. 8), the compound *murein5px4p* (a biomass component) is a product of a reaction whose substrate is *murein5p5p*. Another product of this reaction is *D-Alanine*. When the export reactions of this co-product are removed from the network, the compound *D-Alanine* accumulates and can no longer satisfy the law of conservation of mass. Biomass production, in this pruned network, is impossible: flux-based analysis gives *murein5px4p* a production rate of zero. Nevertheless, removing reactions that export *D-Alanine* has no impact on the graph-based activation of the biomass components. In addition, this compound is not an optimal-efficiency PEM because there are two reactions that can export *D-Alanine*.

In other words, our interpretation is that producibility PEMs that are neither sustainability nor optimal-efficiency PEMs may refer to co-products of essential reactions. They may have a very large high impact on the organism growth rate since their consumption rate is a direct controller of biomass production at steady-state.

In practice, this situation is identified infrequently in all networks except the *Synecchocystis* network: once in the *A. ferrooxidans* and the *T. lutea* networks and two, three and four times in the *iJR*904, *iAF*1260 and *iJO*1366 networks, respectively. This observation may suggest that the co-products have been extensively studied in all of these networks. On the other hand, the *Synecchocystis* network contains 17 producibility PEMs that are neither sustainability nor optimal-efficiency PEMs. This suggests that biomass production is under the control of many co-product export fluxes which merit sensitivity analysis and reflect a need for curation.

##### Producibility and optimal-efficiency-PEM that are not sustainability-PEMs may provide insights on accumulation processes.

This last situation occurs very frequently in the *Synecchocystis*, *A. ferrooxidans* and *T. lutea* networks. Indeed, removing all reactions that consume the PEM in question has no impact on the graph-based activation of the biomass component but, on the contrary, drastically reduces the biomass growth rate by removing an essential reaction. In Fig. 5, both *CO2* and *Ammonia* have this property. The explanation is similar to the one shown in Fig. 8. These PEMs are co-products of essential reactions leading to the production of biomass components. Removing the reactions that consumes these PEMs means that they accumulate in the cell and set the biomass flux rate value to zero, although their topological-based activation is not affected.

## Discussion and Conclusion

In this paper we have introduced a new way of analyzing, describing and differentiating metabolic models in order to gain better insight into their functionality. This method relies on the concept of phenotypic essential metabolites (PEMs), which are key metabolites of the models and can be easily computed using the *Conquests* package. The need for further study of metabolite classification has been highlighted by the lack of differentiation of them performed so far. Apart from key compounds such as seeds (e.g., growth medium) or targets (*e.g.*, biomass components), no in-depth study has been done of the hundreds (or thousands) of remaining compounds, despite their crucial role in network structure. We thus advocate for the determination of PEMs that are cornerstones in the production pathways of target compounds.

Here we have defined three types of PEMs that can be distinguished on the basis of the modeling method used to compute them. Sustainability-PEMs are key components related to the graph-based structure of the graph and the initiation of production paths, whereas producibility and optimal-efficiency-PEMS are related to fluxes distribution at steady-state. The latter focuses on the optimal activation of the target reaction, thus pinpointing metabolites that enable its maximal rate. The computation of sustainability-PEMs was challenging because it relies on a difficult combinatorial problem being solved. The efficiency of the ASP solvers enabled an efficient and fast computation to be produced while allowing all PEMs computations to be contained in a single and easily distributable Python package.

The concept of PEM is related to two concepts of *essential reactions* which are either a reaction whose removal has a lethal effect over the system growth (stoichiometric-based formalism) (Winzeler et al., 1999; Edwards & Palsson, 2000; Duarte, Herrgård & Palsson, 2004; Palumbo et al., 2007; Samal et al., 2006) or a reaction which carries an optimal flux for the biomass production (optimal flux-based formalism) (Gudmundsson & Thiele, 2010). Although the same term of *essential reaction* is used in both cases, it is worth noticing that essential reactions according to an optimal flux-based criteria may not be lethal in the stoichiometric-based framework. According to our formalism, the substrates of the first class of essential reactions (stoichiometric-based formalism) are productibility-PEMs whereas the reactants of the second class of essential reactions (optimal-based framework) are optimal-efficiency-PEMs. PEMs are also related to *essential metabolites* introduced in Kim et al. (2007) and Kim, Kim & Lee (2010), which are metabolites for which the removal of all (multiple) output reactions is lethal, whereas removal of the reactions one by one is not lethal. They also are producibility-PEMs according to our formalism, in addition to the substrates of essential reactions discussed above. To summarize, producibility-PEMs are either reactants of lethal essential reactions (as introduced by Winzeler et al. (1999) according to a stoichiometric-based formalism) or essential metabolites (as introduced by Kim et al. (2007) to take into account multiple reaction deletion), whereas optimal-efficiency-PEMs are reactants of (FVA-based) essential reactions as defined in Gudmundsson & Thiele (2010) according to a FVA-base optimal biomass production criteria. Another related concept is the Minimal Cut Set (Klamt & Gilles, 2004; Beurton-Aimar, Nguyen & Colombie, 2014), which is a set of reactions the removal of which is lethal (stoichiometric-based formalism), although Minimal Cut Sets do not impose any constraints on the substrates of the reactions. Our analysis is that producibility-PEMs are very specific cases of a Minimal Cut Set, since a metabolite is a producibility-PEM if and only if a minimal cut set exists of which the reactions all share the same metabolic compound as a substrate. Sustainability-PEMs are an extension of producibility-PEMs for a graph-based framework. We found no publications in the literature that had pointed them out. To summarize, our concept of phenotypic essential metabolite (PEM) corresponds either to compounds for which the removal of all reactions that consume the compound affects the growth phenotype (sustainability, producibility of biomass) or to compounds for which the removal of a single reaction that consumes the compound affects the growth phenotype (producibility of optimal-efficiency biomass). It allows several concepts to be integrated and compared within a unified framework.

Based on the study of individual examples, we have provided several interpretations of the different situations that may occur with respect to PEMs classification. Sustainability-PEMs provide information about the dependency of production pathways on the initial state of the system. Unlike steady-state-based flux methods, metabolites that are sustainability-PEMs only may enable components to be deciphered which initiate production that can be self-balanced at steady state. Conversely, metabolites that are producibility-PEMs only may indicate pathways that rely on a precise mass-balance equilibrium at steady state. Finally, for synthetic biology purposes, compounds that are optimal-efficiency-PEMs only may be cornerstones as they allow the maximal flux into the reaction of interest. In fact, it seems appropriate to target compounds that might have the biggest impact once altered, to ensure genetic modifications made to a system are effective.

Compounds that are combinations of two types of PEM out of three also reveal interesting features of the network structure. A sustainability and producibility-PEM that is not an optimal-efficiency-PEM may indicate the beginning of alternative production pathways towards targets that are both balanced at steady state and can also be activated in the initial state of the system through a pathway that links the seeds to the PEM. If the sustainability and the optimal-efficiency properties are satisfied but the producibility property is not, a PEM may shed light on internal pathways that are initiated from the graph-based perspective but are not mandatory to activate the target reaction flux. However, those pathways are the only ones that allow the maximal flux to occur. Finally, PEMs that are producibility and optimal-efficiency-PEMs but not sustainability-PEMs may indicate compounds that are carefully balanced in all production pathways that allow the activation of target fluxes. Their consumption, non-accumulation or degradation is essential to prevent mass imbalances whereas graph-based modeling does not take this into account when assessing target activation. One perspective is to work out the terms on which these interpretations, based on phenomena observed in individual examples, can be generalized on the basis of more theoretical studies. As a second perspective, this systematic comparative approach should be applied to multi-scale networks combining regulatory and metabolic features, especially to regulatory FBA (rFBA) formalism (Covert, Schilling & Palsson, 2001).

We conclude that the systematic comparison of several modeling approaches (graph, stoichiometric and optimal flux-based analyses) of the same model may highlight components with different roles within the different formalisms. Our hypothesis is that these compounds often carry relevant information about system dynamics. This promotes the use of the *Conquests* package in two complementary tasks. Either the information about system dynamics is biologically irrelevant and this information may be used for finalizing the curation of genome-scale metabolic networks, or the information about system dynamics is biologically relevant and allows the role of internal cycle and mass-balance equilibria with respect to the production of targeted biomass reactions to be deciphered. Interestingly, the number of PEMs belonging to one or two of the three classes we described is particularly small in genome-scale models that usually contain more than a thousand metabolites. This makes for easier model curation and analysis because those compounds can easily be examined manually.

##  Supplemental Information

10.7717/peerj.3860/supp-1 Supplemental Information 1 Classification of metabolic compounds according to their degree of connectivity (*x*-axis) and their role in the functioning of the network For each interval [a, b] shown on the *x*-axis, the total number of compounds with a degree of connectivity in [a, b] is given at the top of the corresponding bar. The bar is divided into four parts, the height of which is proportional to the number of such compounds classified into the four classes of functioning roles: PEM (blue), seed (i.e. extracellular nutrient) (green), target (i.e biomass component) (red), other generic compounds with no role related to PEMs (yellow). The relative percentages of each class are shown on the *y*-axis. This analysis suggests that the PEM concept not only encompasses highly-connected compounds in a metabolic network but also sheds light on metabolic compounds with a functional role despite their low connectivity.

10.7717/peerj.3860/supp-2 Supplemental Information 2 The different inputs needed to run the Conquests tool on the six metabolic networks studied in the paper

## Additional Information and Declarations

### Data Availability

The following information was supplied regarding data availability: The code sources are available at https://github.com/jlaniau/conquests. The metabolic networks and data used to perform the analyses are available at http://www.irisa.fr/dyliss/public/Conquests/data.zip and have been provided as a Supplemental File.
