# Supplementary figures and images for: Combining graph and flux-based structures to decipher phenotypic essential metabolites within metabolic networks
Source: PeerJ. 2017 Oct 12;5:e3860. doi: 10.7717/peerj.3860 (PMC5641430; doi:10.7717/peerj.3860)

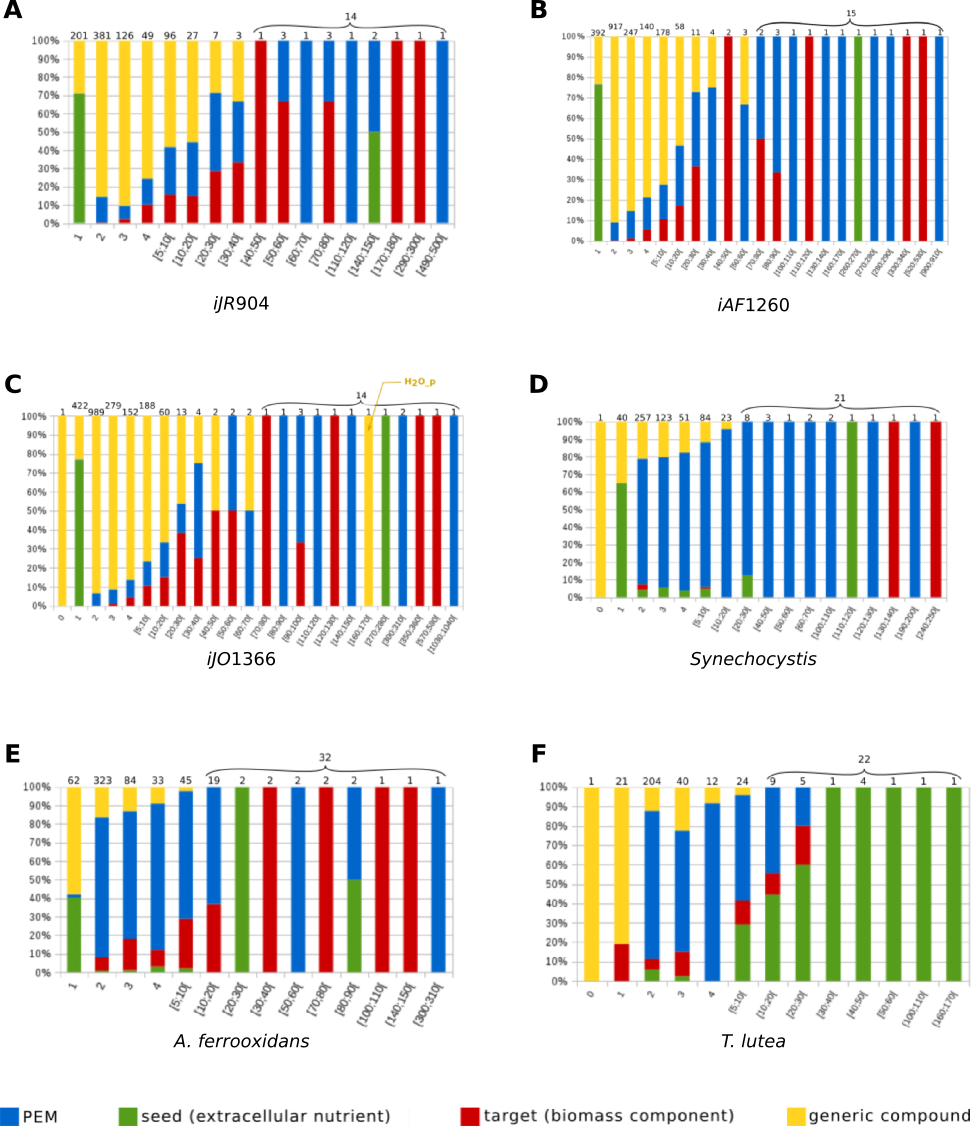

Supplement: Supplemental Information 1 — For each interval [a, b] shown on the x-axis, the total number of compounds with a degree of connectivity in [a, b] is given at the top of the corresponding bar. The bar is divided into four parts, the height of which is proportional to the number of such compounds classified into the four classes of functioning roles: PEM (blue), seed (i.e. extracellular nutrient) (green), target (i.e biomass component) (red), other generic compounds with no role related to PEMs (yellow). The relative percentages of each class are shown on the y-axis. This analysis suggests that the PEM concept not only encompasses highly-connected compounds in a metabolic network but also sheds light on metabolic compounds with a functional role despite their low connectivity. [file peerj-05-3860-s001.png]
